# Changes in symptoms and characteristics of COVID-19 patients across different variants: two years study using neural network analysis

**DOI:** 10.1186/s12879-023-08813-9

**Published:** 2023-11-28

**Authors:** Seyed Hossein Torabi, Seyed Mohammad Riahi, Azadeh Ebrahimzadeh, Fatemeh Salmani

**Affiliations:** 1 School of Medicine, Birjand University of Medical Sciences, Birjand, South Khorasan Province Iran; 2 Epidemiology Department of Family and Community Medicine, School of Medicine Cardiovascular Diseases Research Center, Birjand University of Medical Sciences, Birjand, South Khorasan Province Iran; 3 Department of Infectious Diseases, School of Medicine Infectious Diseases Research Center, Birjand University of Medical Sciences, Birjand, South Khorasan Province Iran; 4 Department of Epidemiology and Biostatistics, School of Health Social Determinants of Health Research Center, Birjand University of Medical Sciences, Birjand, South Khorasan Province Iran

**Keywords:** SARC-COV-2, COVID-19, Strains, Symptoms, Artificial Neural Network

## Abstract

**Background:**

Considering the fact that COVID-19 has undergone various changes over time, its symptoms have also varied. The aim of this study is to describe and compare the changes in personal characteristics, symptoms, and underlying conditions of individuals infected with different strains of COVID-19.

**Methods:**

This descriptive-analytical study was conducted on 46,747 patients who underwent PCR testing during a two-year period from February 22, 2020 to February 23, 2022, in South Khorasan province, Iran. Patient characteristics and symptoms were extracted based on self-report and the information system. The data were analyzed using logistic regression and artificial neural network approaches. The R software was used for analysis and a significance level of 0.05 was considered for the tests.

**Results:**

Among the 46,747 cases analyzed, 23,239 (49.7%) were male, and the mean age was 51.48 ± 21.41 years. There was a significant difference in symptoms among different variants of the disease (*p* < 0.001). The factors with a significant positive association were myalgia (OR: 2.04; 95% CI, 1.76 – 2.36), cough (OR: 1.93; 95% CI, 1.68—2.22), and taste or smell disorder (OR: 2.62; 95% CI, 2.1 – 3.28). Additionally, aging was found to increase the likelihood of testing positive across the six periods.

**Conclusion:**

We found that older age, myalgia, cough and taste/smell disorder are better factors compared to dyspnea or high body temperature, for identifying a COVID-19 patient. As the disease evolved, chills and diarrhea, demonstrated prognostic strength as in Omicron.

**Supplementary Information:**

The online version contains supplementary material available at 10.1186/s12879-023-08813-9.

## Background

In November 2019 a respiratory disease emerged that was later identified to be caused by a new member of the coronaviruses family, a single-stranded RNA virus named SARS-CoV-2 by the International Committee on Taxonomy of Viruses [1, 2]. Since then, the virus that causes COVID-19 has spread to every country in the world [3]. The World Health Organization declared the outbreak a pandemic in February 2020 [4, 5], and the need to break its chain of transmission had become crucial. As SARS-CoV-2 replicates, it can undergo mutations, leading to the emergence of different strains [6]. It is well-established that the epidemiological and clinical features of COVID-19 can vary depending on the predominant strain [7]. In the early days of the outbreak, the disease was characterized mostly by cough, fever and dyspnea [8–10], but as the novel infection grew to inflict upon more people in different regions worldwide, other symptoms emerged as significant in diagnosis including rare symptoms such as changes in smell and taste can complicate the diagnosis of the disease [11]. Six main strains were identified in Iran, namely: the initial strain, B.1.36, B.1.1.413, Alpha, Delta and Omicron. Each variant of the disease has presented with different characteristics. For example, the Omicron variant has shown a higher transmissibility compared to other variants [12]. Investigating the evolution of symptom patterns can assist researchers in understanding the virus's behavior and tracking its progression. There have been studies conducted globally regarding changes in symptom patterns, some of which are referenced in this study [13–16]. The current research conducted within Iranian society, with a substantial number of participants, can provide valuable insights into the evolving symptom patterns within this geographical region. In this study, we aimed at determining the symptoms that predict a positive result on the RT-PCR test in each variant of COVID-19. We took into consideration situations where testing equipment is limited and costly tests need to be reduced. Thus, we only included characteristics observed at admission and did not consider laboratory or other para-clinical findings. This allowed us to identify most probable cases promptly. Our study focused on all variants of COVID-19 that caused an outbreak in Iran, including three Variants of Concern [6]. The goal of our study was to examine the variations in clinical features during periods when different variants were dominant, using artificial intelligence techniques like deep learning and neural network.

## Methods

### Study population and data sources

In this study 46,747 cases were extracted from the hospital information system from 17 hospitals of South Khorasan province, Iran, from the first case identified in the region on 22 February 2020 to 23 February 2022. Over the period, six different variants, including three Variants of Concern (VOC) [6] made an outbreak in the country. The initial outbreak belonged to the novel coronavirus original strain (designated herein as “the Initial variant”) and dominated from the beginning until 3 May 2020 and included 2933 patients. The B.1.36 variant took over from then on until 5 September 2020 and B.1.1.413 from 6 September until 24 January 2021 and there were 5548 and 10,563 suspected patients admitted to the hospitals during these periods, respectively. The Alpha (B.1.1.7) VOC prevailed from 7 September 2021 to 9 June, The Delta (B.1.617.2) VOC from then until 9 January 2022 and at last, Omicron (B.1.1.529) VOC made its outbreak from 10 January 2022 [17, 18] to the date we carried out this study, and the number of the patients comprised 7540, 16,286 and 3877 people, respectively. The diagnoses were confirmed using Reverse Transcription-Polymerase Chain Reactions (RT-PCR) performed on the viral RNA specimens acquired via throat and nasopharyngeal swabs collected from the respiratory tract of the symptomatic individuals. Based on the PCR test results individuals were classified into two groups; SARS‑CoV‑2 positive patients and SARS‑CoV‑2 negative subjects.

Data was registered from history on admission. The characteristics that were recorded comprised of demographic information (sex and age), a number of symptoms (namely, cough, dyspnea, taste or smell disorder, fever, chills, headache, myalgia, sore throat and diarrhoea) and the most prevalent comorbidities (diabetes, cardiovascular diseases, chronic lung diseases, renal diseases, chronic liver diseases, malignancies and immunodeficiency diseases). In this study, fever was defined as having a temperature of ≥ 38°C [19] using Non-contact (Infrared) Thermometers at the admission.

### Statistical analysis

The quantitative variables were described using mean and standard deviation, while the qualitative variables were described using frequency and percentage. The chi-square test was used to compare the proportions in different groups. The independent t-test was used to compare age between the cases with positive and negative test results. The Analysis of Variance test was used to compare the means between different groups. To determine the adjusted associations between reported symptoms and variants of the infection, we utilized multiple logistic regression models with a backward approach. Logistic Regression, a machine learning technique, was presented in the proceedings as a solution for classification problems [20]. Furthermore, the results were validated using a neural network.

We employed powerful multilayer, feed-forward backpropagation, supervised neural networks for prediction and modeling purposes [21]. The neural network structure was determined after testing various combinations of connection functions (hyperbolic tangent or sigmoid for input and middle layer neurons and linear, hyperbolic tangent, or sigmoid functions for middle and output layer neurons), number of hidden layers (one or two), and number of neurons between 2 and 10 in each hidden layer. We used 70% of the data for training and the rest for model evaluation [22]. We supported the results by performing the neural network analyzing for the purpose of determining the importance of association between the factors and the COVID-19 variants. In both approaches, we defined a model for each variant and designated numbers chronologically (1 to 6). The statistical analyses were conducted using the Rattle package of the R statistical software (version 4.1.2) [23]. A significance level of less than 0.05 for the *p*-value was set for all the statistical analyses.

## Results

### Study participants and demographic results

Of a total of 46,747 individuals, 23,239 were men (49.7%) and 23,508 were women, of which the PCR test for COVID-19 was positive for 15,626 (33.4%) patients. Among PCR positive patients 49.5% were male and 50.5% female. Patients were on average 51.48 ± 21.41 years (across different variants) and SARS-2 negative patients were 46.39 ± 26.08 years of age. Out of the SARS-CoV-2 positive group, 8% of the individuals passed away, whereas, the percentage of deaths in the negative group was only 1.1. According to Table 1, the mean age of patients with the Omicron and Delta variants was significantly lower than other variants. In all studied symptoms except for malignancy and immunodeficiency, there is a significant difference between strains (*P* < 0.05).

**Table 1:** Between variants comparison of demographic characteristics and symptoms in people with positive test results in different variants

Variables		**Initial** **(*n* = 547)**	**B.1.36** **(*n* = 1262)**	**B.1.1.413** **(*n* = 5418)**	**Alpha (*n* = 1806)**	**Delta** **(*n* = 5025)**	**Omicron (*n* = 1568)**	*P***-value**
**Age**		49.00 ± 22.37	48.42 ± 23.94	48.42 ± 23.43	49.19 ± 26.05	46.44 ± 25.09	44.33 ± 26.37	< 0.001
**Sex**	Male	1638 (55.8)	2856 (51.5)	5424(51.3)	3685(48.9)	7813(48)	1823(47)	< 0.001
	Female	1295(44.2)	2692(48.5)	5139(48.7)	3855(51.1)	8473(52)	2054(53)	
**Comorbidities**								
**Diabetes**		73(13.8)	131(10.5)	510(9.4)	182(10.1)	366(7.3)	101(6.3)	< 0.001
**Chronic lung disease**		25(4.7)	28(2.2)	145(2.7)	58(3.2)	107(2.1)	37(2.3)	0.003
C**ardiovascular disease**		117(22.1)	213(17.1)	863(15.9)	305(16.9)	641(12.8)	177(11.1)	< 0.001
**Renal disease**		20(3.8)	21(1.7)	70(1.3)	16(0.9)	63(1.3)	23(1.4)	0.017
**Chorionic Liver disease**		8(1.5)	6(0.5)	0	0	0	0	< 0.001
**Malignancies**		4(0.8)	9(0.7)	32(0.6)	8(0.4)	28(0.6)	10(0.6)	0.884
**Immunodeficiency**		0	8(0.6)	21(0.4)	6(0.3)	20(0.4)	10(0.6)	0.322
**Signs and symptoms**								
C**ough**		279(52.6)	483(38.7)	2467(45.4)	811(45)	2097(41.8)	664(41.6)	< 0.001
**Chills**		0	0	0	259(14.4)	1036(20.7)	300(18.8)	< 0.001
**Myalgia**		126(23.8)	407(32.6)	2402(44.2)	657(36.4)	2199(43.9)	708(44.4)	< 0.001
**Taste/smell disorder**		0	0	0	33(1.8)	193(3.8)	35(2.2)	< 0.001
**Diarrhoea**		23(4.3)	96(7.7)	327(6)	69(3.8)	189(3.8)	31(1.9)	< 0.001
**Dyspnea**		210(39.6)	463(37.1)	2172(40)	812(45)	2101(41.9)	540(33.8)	< 0.001
**Headache**		90(17)	252(20.2)	1361(25.1)	303(16.8)	1077(21.5)	388(24.3)	< 0.001
**Sore Throat**		49(9.2)	127(10.2)	633(11.7)	214(11.9)	657(13.1)	460(28.8)	< 0.001
**Fever**		102(19.2)	213(17.1)	1361(25.1)	361(20)	1101(22)	231(14.5)	< 0.001

### Symptom presentation and univariate analysis across variants

Based on univariate analysis of variants, age variable was significantly higher in cases with a positive test result in all variants. Generally, fever has been a common symptom among individuals with and without a positive test result. However, the odds for running a fever in the B.1.1.413 variant, were more in favor of patients with positive test results, whereas in the Omicron variant, patients with a negative test result had higher chances of having a fever (Table 2). Cough is an important predictor of COVID-19 in all variants expect for Omicron. The only symptom that showed strong significant results in favor of having each variant was myalgia (*p* < 0.001). Dyspnea is seen more in patients with a positive test result in variants B1.36, B.1.1.413 and Alpha compared to PCR-negative patients. In Alpha, Delta and Omicron variants, a significant incidence of smell or taste disorder was observed in PCR-positive patients. Also, the prevalence of chills, myalgia and smell or taste disorder was observed more in patients with the Delta and Omicron variants (*P* < 0.001). The presence of diarrhoea did not result in a positive test in all variants.

**Table 2:** Within variants comparison of demographic characteristics and symptoms

Characteristics	Initial (*n* = 2933)			B.1.36 (*n* = 5548)			B.1.1.413 (*n* = 10,563)			Alpha (*n* = 7540)			Delta (*n* = 16,286)			Omicron (*n* = 3877)		
	RT-PCR + (*n* = 547)	RT-PCR – (*n* = 2386)	*p*	RT-PCR + (*n* = 1262)	RT-PCR – (*n* = 4286)	*p*	RT-PCR + (*n* = 5418)	RT-PCR – (*n* = 5145)	*p*	RT-PCR + (*n* = 1806)	RT-PCR – (*n* = 5734)	*p*	RT-PCR + (*n* = 5025)	RT-PCR – (*n* = 11,261)	*p*	RT-PCR + (*n* = 1568)	RT-PCR – (*n* = 2309)	*p*
*Demographics*																		
Age (yrs.), mean ± SD	51.7 ± 20	48.7 ± 22.9	**0.003**	52.1 ± 20.2	47.8 ± 24.9	** < 0.001**	52 ± 21.1	46 ± 25.3	** < 0.001**	56 ± 21.2	47.6 ± 27.1	** < 0.001**	50.3 ± 20.8	45.5 ± 26.6	** < 0.001**	47.6 ± 24.8	43.6 ± 27.3	** < 0.001**
Sex																		
Male (%)	323 (59)	1315 (55.1)	0.094	618 (49)	2238 (55.2)	**0.043**	2814 (51.9)	2610 (50.7)	0.214	836 (46.3)	2849 (49.7)	**0.012**	2408 (47.9)	5405 (48)	0.928	730 (46.6)	1093 (47.3)	0.633
Female (%)	224 (41)	1071 (44.9)		644 (51)	2048 (47.8)		2604 (48.1)	2535 (49.3)		970 (53.7)	2885 (50.3)		2617 (52.1)	5856 (52)		838 (53.4)	1216 (52.7)	
*Comorbidities*																		
Diabetes	73 (13.3)	159 (6.7)	** < 0.001**	131 (10.4)	327 (7.6)	**0.002**	511 (9.4)	387 (7.5)	** < 0.001**	183 (10.1)	183 (7.6)	**0.001**	367 (7.3)	762 (6.8)	0.213	98 (6.3)	136 (5.9)	0.644
Chronic lung disease	25 (4.6)	190 (8)	**0.013**	29 (2.3)	219 (5.1)	** < 0.001**	145 (2.6)	214 (4.1)	** < 0.001**	57 (3.2)	298 (5.2)	**0.002**	108 (2.1)	473 (4.2)	** < 0.001**	37 (2.3)	123 (5.3)	** < 0.001**
Cardiovascular disease	117 (21.4)	377 (15.8)	**0.002**	216 (17.1)	668 (15.5)	0.274	864 (15.9)	739 (14.4)	**0.001**	304 (16.9)	899 (15.7)	0.1	644 (12.8)	1385 (12.3)	**0.009**	171 (10.9)	270 (11.7)	**0.041**
Renal disease	20 (3.7)	86 (3.6)	0.953	21 (1.7)	94 (2.2)	0.246	70 (1.3)	93 (1.8)	**0.032**	16 (0.9)	127 (2.2)	** < 0.001**	63 (1.3)	209 (1.9)	**0.006**	23 (1.5)	35 (1.5)	0.902
Chronic liver disease	8 (1.5)	13 (0.5)	**0.022**	6 (0.5)	21 (0.5)	0.167	0 (0)	2 (0)	0.237	0 (0)	0 (0)	-	0 (0)	0 (0)	-	0 (0)	0 (0)	-
Malignancies	4 (0.7)	47 (2)	0.046	9 (0.7)	82 (1.9)	**0.006**	32 (0.6)	60 (1.2)	**0.003**	9 (0.5)	62 (1.1)	0.078	27 (0.5)	90 (0.8)	0.105	10 (0.6)	18 (0.7)	0.395
Immunodeficiencies	1 (0.2)	9 (0.4)	0.482	8 (0.6)	27 (0.6)	0.988	20 (0.4)	39 (0.8)	**0.007**	6 (0.3)	69 (1.2)	**0.001**	20 (0.4)	135 (1.2)	** < 0.001**	10 (0.6)	33 (1.4)	**0.021**
*signs and symptoms*																		
Cough	279 (51)	926 (38.8)	** < 0.001**	494 (39.1)	990 (23.1)	** < 0.001**	2462 (45.4)	1746 (33.9)	** < 0.001**	811 (44.9)	1741 (30.4)	** < 0.001**	2103 (41.9)	3293 (29.2)	** < 0.001**	652 (41.6)	911 (39.5)	0.185
Chills	0 (0)	0 (0)	-	0 (0)	0 (0)	-	0 (0)	0 (0)	-	263 (14.6)	532 (9.3)	** < 0.001**	1039 (20.7)	1544 (13.7)	** < 0.001**	293 (18.7)	294 (12.7)	** < 0.001**
Myalgia	127 (23.2)	322 (13.5)	** < 0.001**	416 (33)	782 (18.2)	** < 0.001**	2395 (44.2)	1718 (33.4)	** < 0.001**	664 (36.8)	1717 (29.9)	** < 0.001**	2200 (43.8)	4465 (39.7)	** < 0.001**	697 (44.5)	821 (35.6)	** < 0.001**
Taste/smell disorder	0 (0)	0 (0)	-	0 (0)	0 (0)	-	0 (0)	0 (0)	-	33 (1.8)	52 (0.9)	**0.001**	194 (3.9)	150 (1.3)	** < 0.001**	34 (2.2)	26 (1.1)	**0.01**
Diarrhoea	24 (4.4)	119 (5)	0.557	97 (7.7)	398 (9.3)	0.08	327 (6)	420 (8.2)	** < 0.001**	67 (3.7)	503 (8.8)	** < 0.001**	191 (3.8)	757 (6.7)	** < 0.001**	29 (1.8)	108 (4.7)	** < 0.001**
Dyspnea	211 (38.6)	890 (37.3)	0.579	473 (37.5)	1285 (30)	** < 0.001**	2170 (40.1)	1742 (33.9)	** < 0.001**	807 (44.7)	1968 (34.3)	** < 0.001**	2106 (41.9)	3850 (34.2)	** < 0.001**	531 (33.9)	833 (36.1)	0.157
Headache	91 (16.6)	339 (14.2)	0.148	252 (20)	553 (12.9)	** < 0.001**	1363 (25.2)	1077 (20.9)	** < 0.001**	302 (16.7)	832 (14.5)	**0.022**	1085 (21.6)	2050 (18.2)	** < 0.001**	378 (24.1)	481 (20.8)	**0.016**
Sore Throat	49 (9)	288 (12.1)	**0.04**	131 (10.4)	302 (7)	** < 0.001**	630 (11.6)	594 (11.5)	0.894	214 (11.8)	603 (10.5)	0.112	658 (13.1)	1496 (13.3)	0.741	458 (29.2)	453 (19.6)	** < 0.001**
Fever^a^	102 (18.6)	380 (15.9)	0.121	225 (17.8)	758 (17.7)	0.907	1352 (25)	1093 (21.2)	** < 0.001**	362 (20)	1202 (21)	0.401	1101 (21.9)	2571 (22.8)	0.194	227 (14.5)	432 (18.7)	**0.001**

Data are presented as n (%); the percentage is expressed as within the variant

*Abbreviations*: *RT-PCR* Reverse Transcription-Polymerase Chain Reaction; *yrs.* years, *SD* Standard Deviation, *p* *p*-value

^a^Fever is defined as a body temperature ≥ 38 °C, The bolded values are significant

In relation to underlying conditions, diabetes, chronic lung disease, cardiovascular, renal and chorionic liver diseases were more prevalent in the early variants. The presence of chronic lung and heart diseases was significantly higher in most variants among cases with a negative test result. Diabetes was a significant risk factor in the primary variants (B.1.36, B.1.1.413 and Alpha), while there was no significant difference in the two groups with positive and negative test results in the Delta and Omicron variants (Table 2).

### Multivariate analysis by machine learning logistic regression approach

Based on the machine learning modeling logistic regression, The most prominent positive associations were myalgia (OR: 2.03; 95% CI, 1.6 – 2.57; *p* < 0.001, Model 1 (the initial); OR: 2.04; 95% CI, 1.76 – 2.36; *p* < 0.001, Model 2 (B.1.36)), cough (OR: 1.93; 95% CI, 1.68—2.22; *p* < 0.001, Model 2; OR: 1.81; 95% CI, 1.62—2.03; *p* < 0.001, Model 4 (Alpha)), taste or smell disorder (OR: 2.62; 95% CI, 2.1 – 3.28; *p* < 0.001, Model 5 (Delta)), headache (OR: 1.51; 95% CI, 1.26—1.79; *p* < 0.001, Model 2), chills (OR: 1.7; 95% CI, 1.43 – 2; *p* < 0.001, Model 4; OR: 1.71; 95% CI, 1.43 – 2.04; *p* < 0.001, Model 6 (Omicron)) and sore throat (OR: 1.6; 95% CI, 1.37 – 1.86; *p* < 0.001, Model 6). On the other hand there were also a few significant negative associations: sore throat (OR: 0.6; 95% CI, 0.43 – 0.84; *p* = 0.003, Model 1), diarrhoea (OR: 0.48; 95% CI, 0.32 – 0.73; *p* = 0.001, Model 6; OR: 0.62; 95% CI, 0.47 – 0.81; *p* = 0.001, Model 4) and surprisingly fever in the Omicron outbreak (OR: 0.78; 95% CI, 0.65 – 0.94; *p* = 0.009). In demographics, aging (for each 10 years older) showed 6–12% likelihood of returning a positive test across the six periods (Table 3).

**Table 3:** Estimation of the simultaneous odds ratio of predictive symptoms for different COVID-19 strains based on a multiple regression model

**Models**	1: Initial		2: B.1.36		3: B.1.1.413		4: Alpha		5: Delta		6: Omicron	
**Variable**	OR (95% CI)	*p*	OR (95% CI)	*p*	OR (95% CI)	*p*	OR (95% CI)	*p*	OR (95% CI)	*p*	OR (95% CI)	*p*
Age	1.07 (1.03–1.12)	0.001	1.08 (1.05–1.11)	< 0.001	1.12 (1.10–1.14)	< 0.001	1.12 (1.10–1.15)	< 0.001	1.06 (1.05–1.08)	< 0.001	1.06 (1.04–1.09)	< 0.001
Cough	1.72 (1.42–2.09)	< 0.001	1.93 (1.68–2.22)	< 0.001	1.68 (1.55–1.82)	< 0.001	1.81 (1.62–2.03)	< 0.001	1.65 (1.54–1.78)	< 0.001	**…**	
Sore Throat	0.6 (0.43–0.84)	0.003	**…**		**…**		**…**		**…**		1.6 (1.37–1.86)	< 0.001
Taste/smell disorder	**…**		**…**		**…**		1.66 (1.04–2.64)	0.03	2.62 (2.1–3.28)	< 0.001	**…**	
Myalgia	2.03 (1.6–2.57)	< 0.001	2.04 (1.76–2.36)	< 0.001	1.72 (1.58–1.87)	< 0.001	1.51 (1.34–1.70)	< 0.001	1.26 (1.17–1.35)	< 0.001	1.4 (1.23–1.6)	< 0.001
Headache	**…**		1.51 (1.26 -1.79)	< 0.001	1.31 (1.19–1.44)	< 0.001	1.21 (1.04–1.41)	0.01	1.22 (1.12–1.33)	< 0.001	**…**	
Dyspnea	**…**		1.18 (1.02–1.36)	0.02	1.26 (1.16–1.37)	< 0.001	1.4 (1.24–1.57)	< 0.001	1.28 (1.19–1.38)	< 0.001	**…**	
Fever	**…**		**…**		1.4 (1.27–1.54)	< 0.001	**…**		**…**		0.78 (0.65–0.94)	0.009
Diarrhea	**…**		**…**		0.85 (0.73–1.0)	0.05	0.62 (0.47–0.81)	0.001	0.65 (0.55–0.77)	< 0.001	0.48 (0.32–0.73)	0.001
Chill	**…**		**…**		**…**		1.7 (1.43–2)	< 0.001	1.59 (1.45–1.74)	< 0.001	1.71 (1.43–2.04)	< 0.001
Sex (Female)	**…**		**…**		**…**		1.13 (1.02–1.27)	0.02	**…**		**…**	

*Abbreviations*: *OR* Odds Ratio, *CI* Confidence Interval

### Determining the importance of predictive symptoms by Artificial Neural Network (ANN)

Eighteen independent variables of 46,747 total patients were used to build the ANN. The output classes were positive and negative RT-PCR test, for each variant. As a result, age, sore throat, myalgia, along cough and diarrhoea with an accuracy of 81.7%, were the most important factors in the model evaluating the Initial strain (Table 4). The importance of age increased in subsequent variants and remained on top. The most prominent predictors besides age were myalgia in B.1.36 (model accuracy: 77.3%); cough, myalgia, fever in B.1.1.413 (59.6%); diarrhoea, and to a lesser extent, taste/smell disorder in Alpha (75.7%); taste or smell disorder, cough, and diarrhoea in Delta (69.2%) and, chill and diarrhoea in Omicron (62.5%) (For more details see Supplementary) (Table 4).

**Table 4:** Estimation of the importance level of symptoms as predictors of disease for each strain based on a neural network model

Strains	Initial		B.1.36		B.1.1.413		Alpha		Delta		Omicron	
**Accuracy**	81.7%		77.3%		59.6%		75.7%		69.2%		62.5%	
	Predictors	Percent	Predictors	Percent	Predictors	Percent	Predictors	Per cent	Predictors	Percent	Predictors	Percent
	Age	30	Age	45	Age	40	Age	37	Age	40	Age	27
	Sore throat	20	Myalgia	20	Cough	18	Diarrhoea	18	Smell disorder	18	Chills	19
	Myalgia	19	Cough	13	Myalgia	17	smell disorder	16	Cough	16	Diarrhoea	14
	Cough	17	Headache	7	Fever	16	Headache	15	Diarrhoea	14	Sore throat	12

## Discussion

The study analyzed a vast amount of data from inpatient populations who exhibited signs and symptoms indicative of COVID-19 upon admission. The objective was to identify the most predictive characteristics associated with each SARS-CoV-2 variant responsible for causing outbreaks in Iran and South Khorasan province over a two-year period. During this time, there were a total of six outbreaks attributed to six distinct strains of SARS-CoV-2,

Of particular significance, our analysis found that fever (defined as a temperature of ≥ 38°C), which has been commonly included in a triad of symptoms (alongside dyspnea and cough) used to diagnose COVID-19 [8, 9, 24], was only significantly associated with the B.1.1.413 variant. Conversely, we found a significant negative association between fever and the Omicron coronavirus variant. In a study conducted by Mousavi et al., normal body temperature was observed in patients during the period corresponding to the first five waves of our study [25]. However, the absence of fever in these patients could be attributed to the possible use of antipyretics prior to seeking medical attention.

We included underlying diseases only in the univariate analyses. The results for most of the comorbidities in our study were against the expectation especially for chronic lung disease, as they were shown to be more common among SARS-CoV-2 PCR negative patients. There appears to be an inverse relationship between chronic lung disease and the likelihood of testing positive for COVID-19. Godbout et al. showed that people with underlying diseases maintained lower levels of contacts as they perceived themselves at risk of COVID-19 complications [26, 27]. Guntur et al. found that chronic respiratory disease (asthma, ILD and COPD) were not associated with SARS-CoV-2 PCR positive test; Roland et al. also saw chronic lung disease more uncommon among COVID-19 positives [28–31]. Diabetes was identified as a significant risk factor for the primary variants of SARS-CoV-2, but it does not appear to be a major risk factor for the Delta and Omicron variants.

Myalgia, age (with an increase in risk for every ten years of age), and cough were consistently identified as significant characteristics across all variants. Notably, the probability of receiving a positive PCR test result decreased with the presence of myalgia as SARS-CoV-2 progressed over time; this trend was particularly evident in the B.1.36 and initial variants, and less so in later variants. In contrast, the association between aging and a positive test result was mostly increasing over time. Although cough had a higher prognostic value based on adjusted odds ratios, age was consistently identified as the most important characteristic across all six variants according to neural network analyses. These two symptoms, along with older age, have been frequently reported as signs of COVID-19 in various studies [32–39]. It is noteworthy that dyspnea was not found to be a significant factor at the onset of the pandemic either. This may be attributed to the fact that our data was limited to the time of admission, while dyspnea typically develops later in the course of the disease.

Loss of smell or taste has been reported by multiple studies as one of the most specific symptoms associated with SARS-CoV-2 positivity [8, 11, 28, 40–44] In our study, when sufficient data was available, such as during the Delta and Alpha periods, taste or smell disorders exhibited strong associations in our machine learning models, which were further supported by neural network analyses. Vihta et al. also reported a strong association between loss of taste/smell and COVID-19. They found that the reporting of loss of taste/smell was highest during the Delta period, followed by the wild-type and Alpha strain [43]. Due to a lack of public awareness, we did not have registered data for taste/smell disorders during the first three waves of our study. Hawkes has suggested that the actual prevalence of taste/smell impairment may be much higher than what is being reported by patients [11]. Other studies have reported that loss or change of taste/smell was indicative of the Alpha strain [45, 46] and additionally have confirmed its lower predictive strength compared to the Delta strain [44].

Sore throat and fever were the only symptoms in our study that showed inconsistent associations across the different variants, being significant in only two variants each. According to our Neural network modeling, sore throat was the second most important factor after age in the Initial variant. Of note, whereas sore throat acted to be statistically significant for predicting Omicron, taste/smell impairment did not return such relation for Omicron after adjustment for other factors in machine-learning modeling. This finding is consistent with some studies that have reported an increase in sore throat and a reduction in taste/smell disorder in Omicron cases [44, 47–49].

In this study, headache was found to be significant during the second surge of the disease (B.1.36), but its predictive strength decreased as the virus evolved, and it was no longer significant in Omicron. In Omicron, headache -similar to taste/smell disorder- showed a meaningful difference between COVID-19 and non-COVID-19 patients in univariate analysis, but after adjusting by other factors in the multivariate modeling, headache did not show up in the results for Omicron. This is consistent with other studies that have reported headache as a less significant symptom in Omicron [44, 50]. For instance, Ekroth found that although crude proportions suggested similar rates of headache between Delta and Omicron, after adjustment, headache was in favor of Delta infections [48].

Diarrhoea, a gastrointestinal symptom present from the early weeks of the pandemic, was initially reported to be more common among PCR-negative individuals [8, 9, 51], However, it has gradually emerged as a significant characteristic in the last three variants, and is now considered a negative predictor of COVID-19 [47, 48, 52].

Based on our two approaches—Machine-learning logistic regression and Neural network modeling, we have identified significant predictors for the variants evaluated in this study. For the initial variant, older age (for every 10 years), sore throat (negatively), myalgia, and cough were identified as significant predictors. For the B.1.36 variant, aging, myalgia, cough, and headache were significant predictors. In the B.1.1.413 variant, age, cough, myalgia, fever, and headache were significant predictors. For Alpha, age, diarrhoea (negatively), taste or smell impairment, cough, myalgia, headache, and chills were significant predictors. For the Delta strain, aging, taste or smell disorder, cough, and diarrhoea (negatively) were significant predictors, while for the Omicron variant, age, chills, diarrhoea (negatively), and sore throat were significant predictors.

Overall, age, myalgia, cough, and taste or smell disorder were identified as the most reliable factors to predict a positive test result for the first five variants. However, for the Omicron variant, chills and not having diarrhoea were found to be more decisive than cough or myalgia. It's worth noting that the Omicron variant, which is still being observed as a circulating VOC, is atypical compared to previous variants [6]. The supposed three common symptoms for COVID-19, namely cough, fever, and dyspnea, did not even show up as predictors for the Omicron variant. This change of symptoms in Omicron [49, 53] can be linked to the vaccine effect [47], although we did not consider the vaccination status in our study.

In conclusion, our study provides valuable insights into the symptomatology of different COVID-19 variants and could be useful for early diagnosis and management of the disease.

To the best of our knowledge, this paper is one of the few studies to date that has examined multiple COVID-19 variants and their clinical characteristics using multivariable modeling. In addition to the modeling approaches, this study’s key strength includes its large cohort and the fact that RT-PCR tests were performed for anyone with suspected COVID-19 or respiratory disease symptoms, regardless of their chief complaint. However, our study had some limitations. First, the symptoms collated were mostly self-reported. Second, there was no RT-PCR testing equipment available exclusively for each strain, so we differentiated them based on the periods of time when each variant was dominant. It should be noted that, due to the accuracy of the testing equipment, there were subjects with false-negative PCR results who were treated as COVID-19 patients based on other diagnostic methods, such as CT scans of the lungs and clinicians' judgment. However, in this study, they were considered non-COVID patients.

## Conclusion

The present study aimed to investigate the clinical symptoms, comorbidities, and demographics of all symptomatic patients suspected of COVID-19 in the South Khorasan province from the emergence of the disease to the time this study was undertaken. The results indicate that older age, myalgia, cough, and taste or smell disorder are better predictors of COVID-19 than dyspnea or high body temperature. As the disease evolves, symptoms such as chills and diarrhoea demonstrate prognostic strength, as in the case of Omicron. These findings can be used to stratify next steps for isolation or hospitalization based on patient’s clinical conditions and to trace back in-contact subjects. It is important to be vigilant not to miss patients based on classic signs and symptoms, and this study could be beneficial for both the healthcare professionals and the general public in early recognition of COVID-19 infection.

### Supplementary Information

**Additional file 1. **Estimation of the importance level of symptoms as predictors of disease for each strain based on a neural network model.

## Data Availability

The datasets used and analysed during the current study are available from the corresponding author on reasonable request.
