# Supplementary material for: Changes in symptoms and characteristics of COVID-19 patients across different variants: two years study using neural network analysis
Source: BMC Infect Dis. 2023 Nov 28;23:838. doi: 10.1186/s12879-023-08813-9 (PMC10683353; doi:10.1186/s12879-023-08813-9)
Supplement: Supplementary file 1 — Additional file 1. Estimation of the importance level of symptoms as predictors of disease for each strain based on a neural network model. [file 12879_2023_8813_MOESM1_ESM.docx]

| Estimation of the importance level of symptoms as predictors of disease for each strain based on a neural network model | |
| --- | --- |
| Initial | 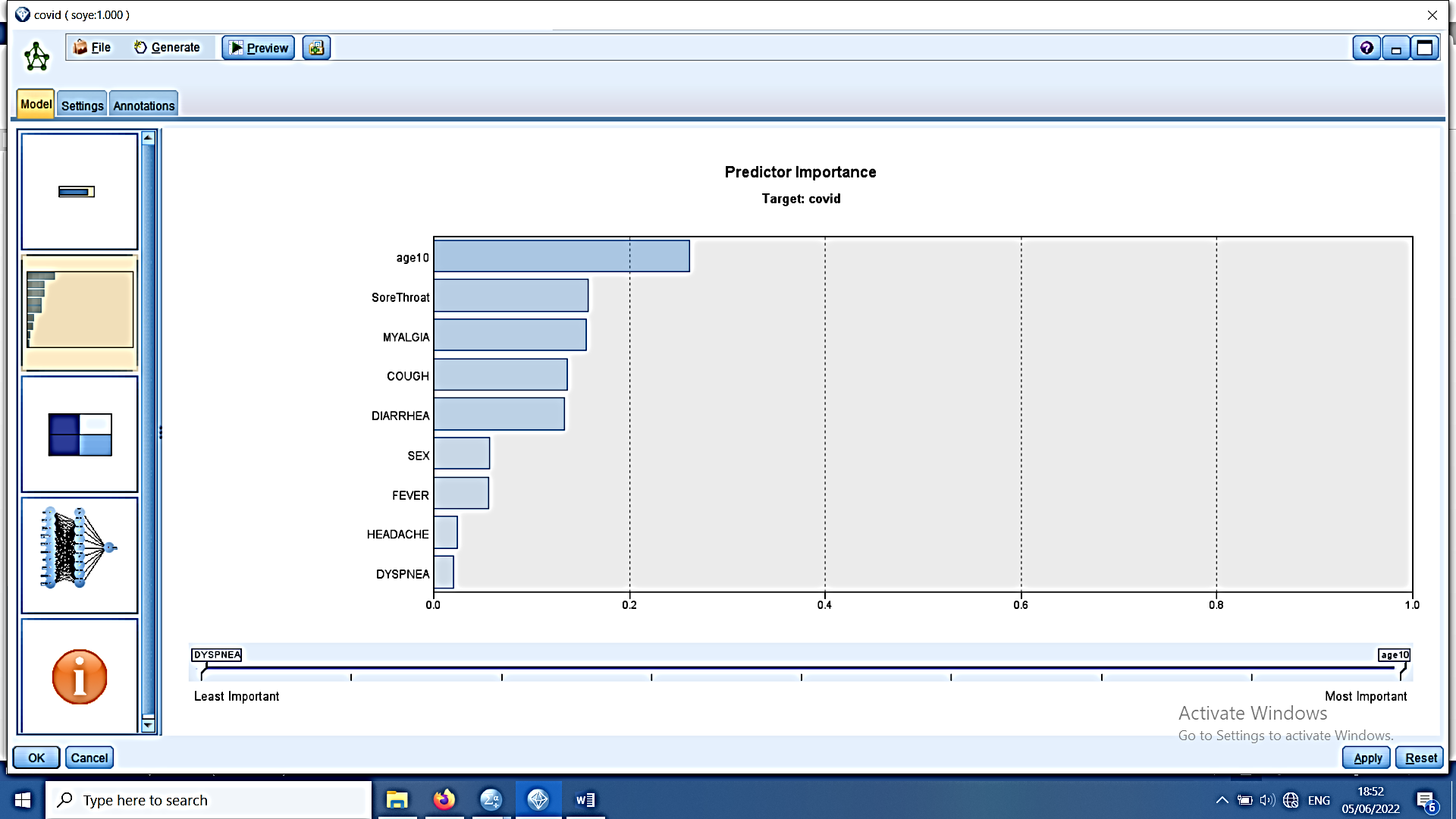 |
| B.1.36 | 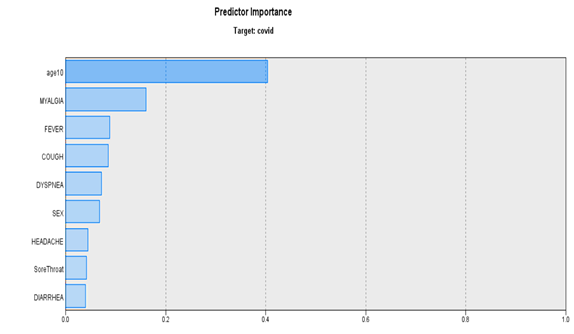 |
| B.1.1.413 | 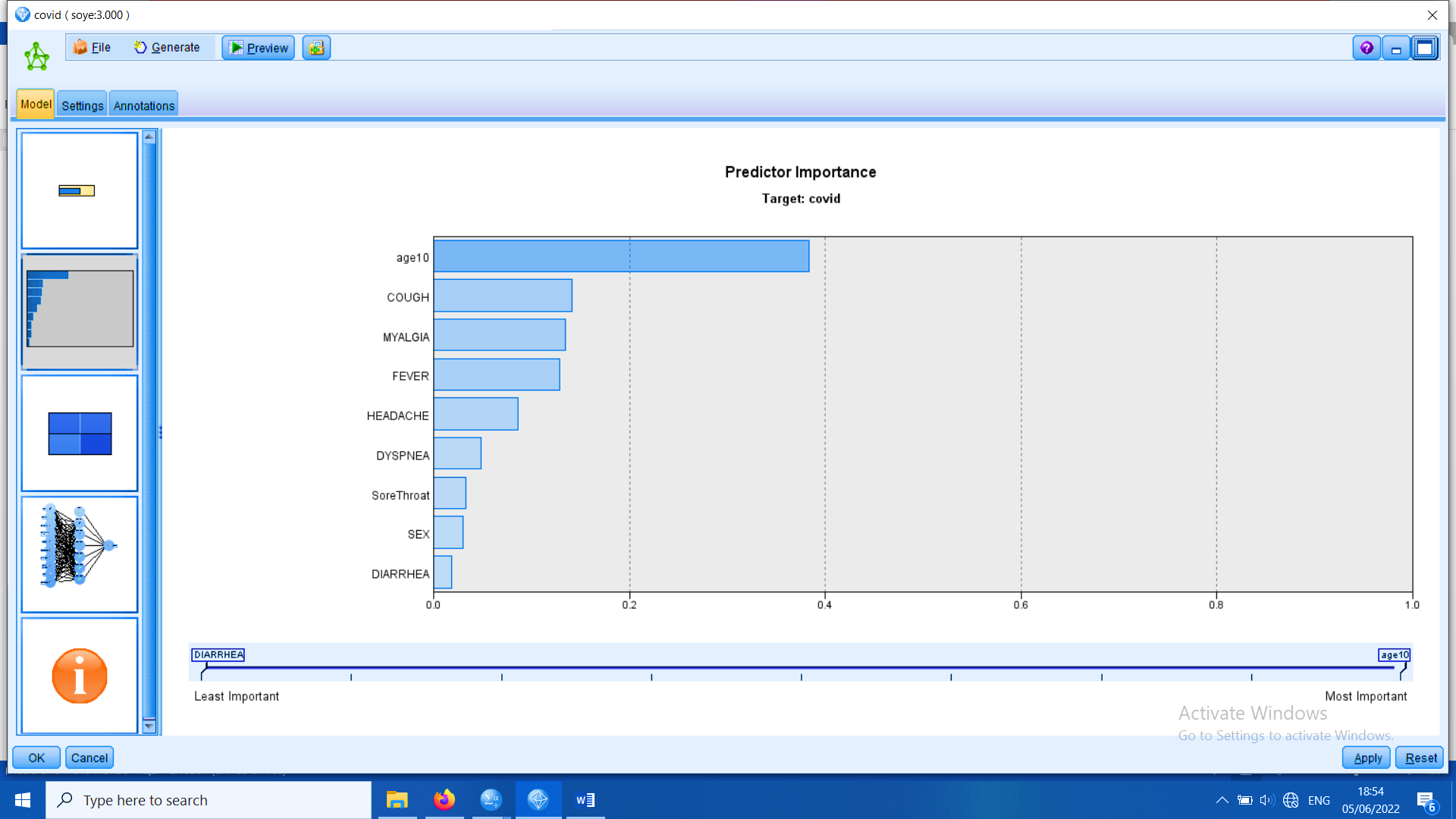 |
| Alpha | 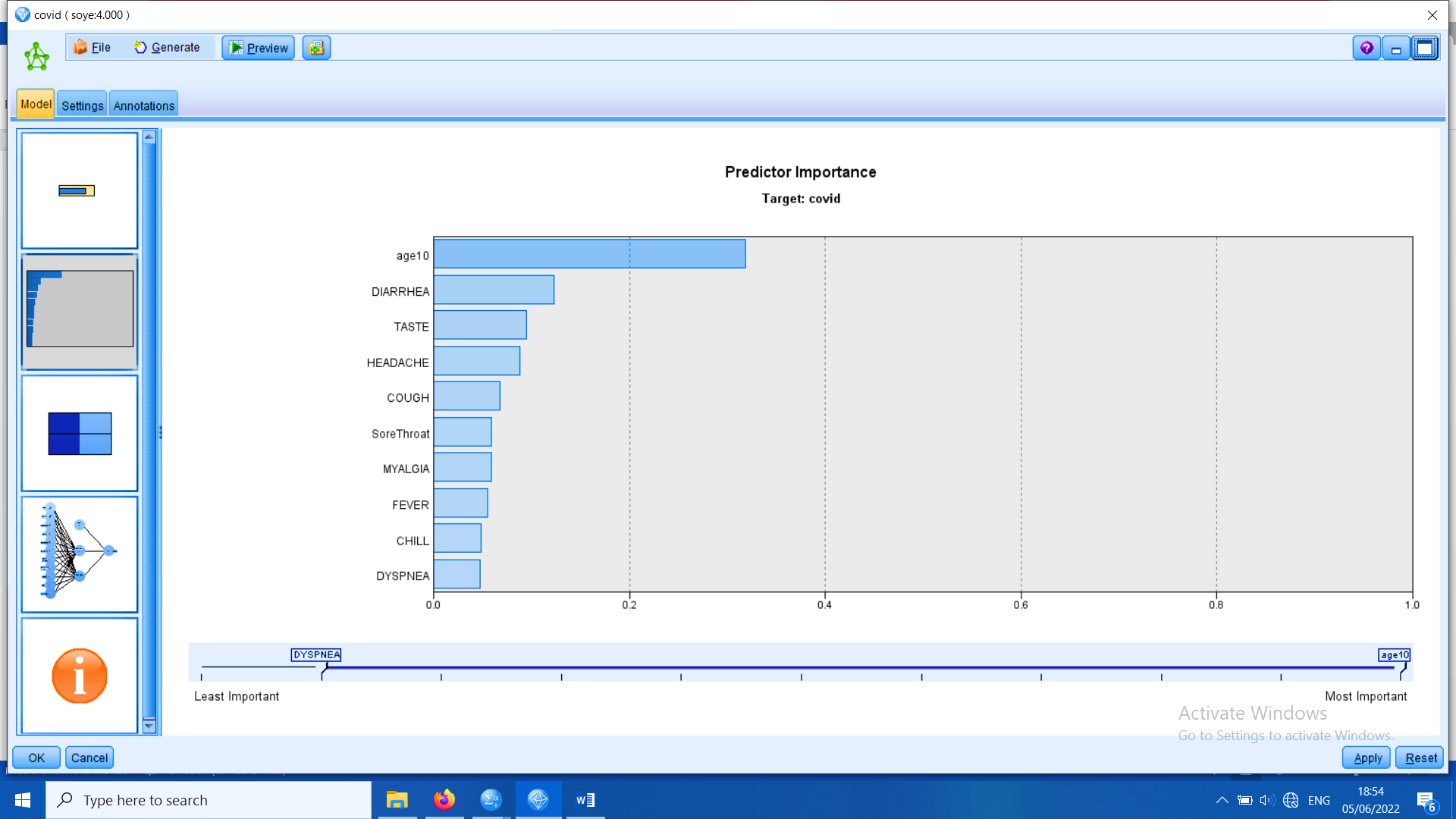 |
| Delta | 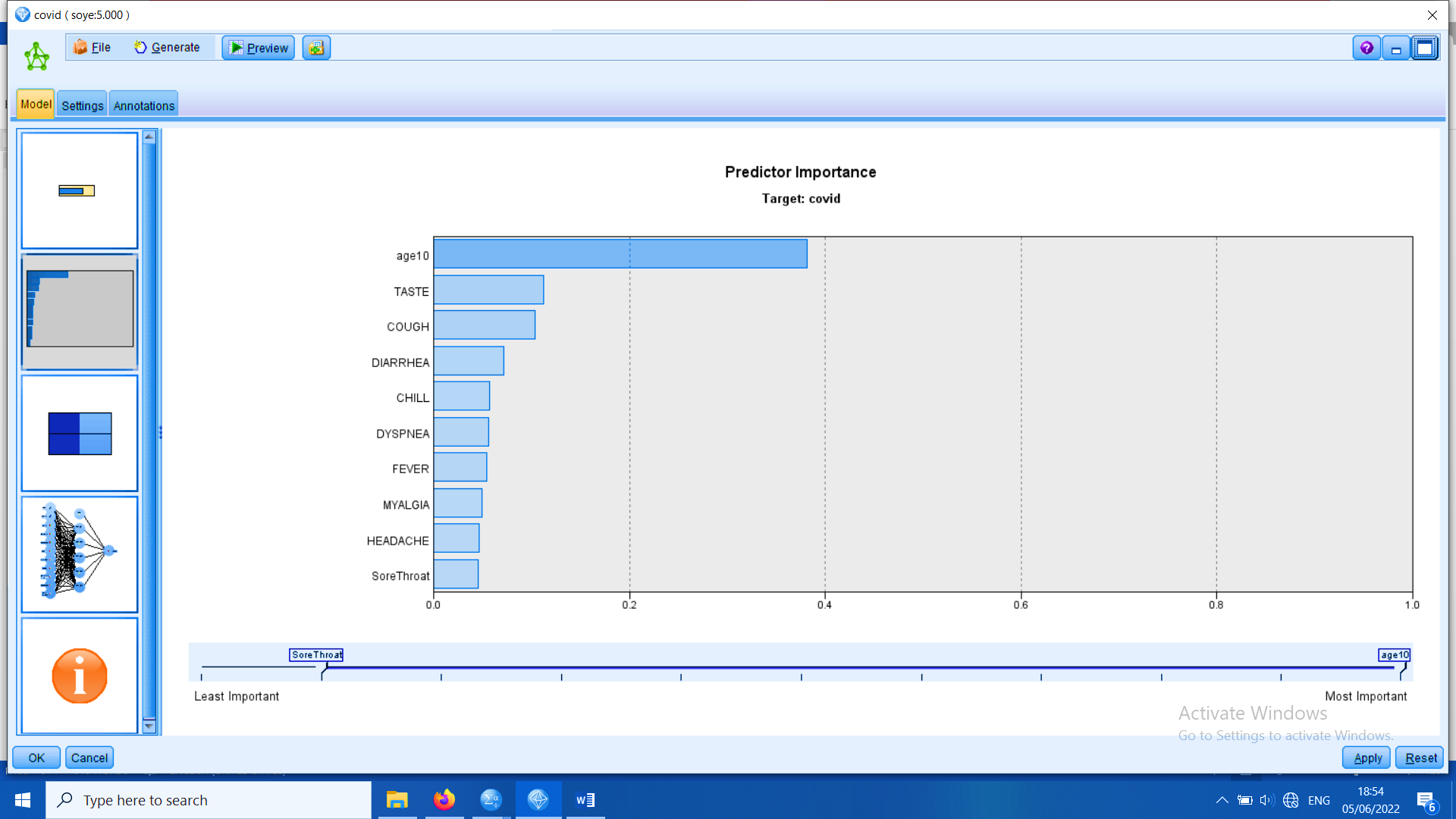 |
| Omicron | 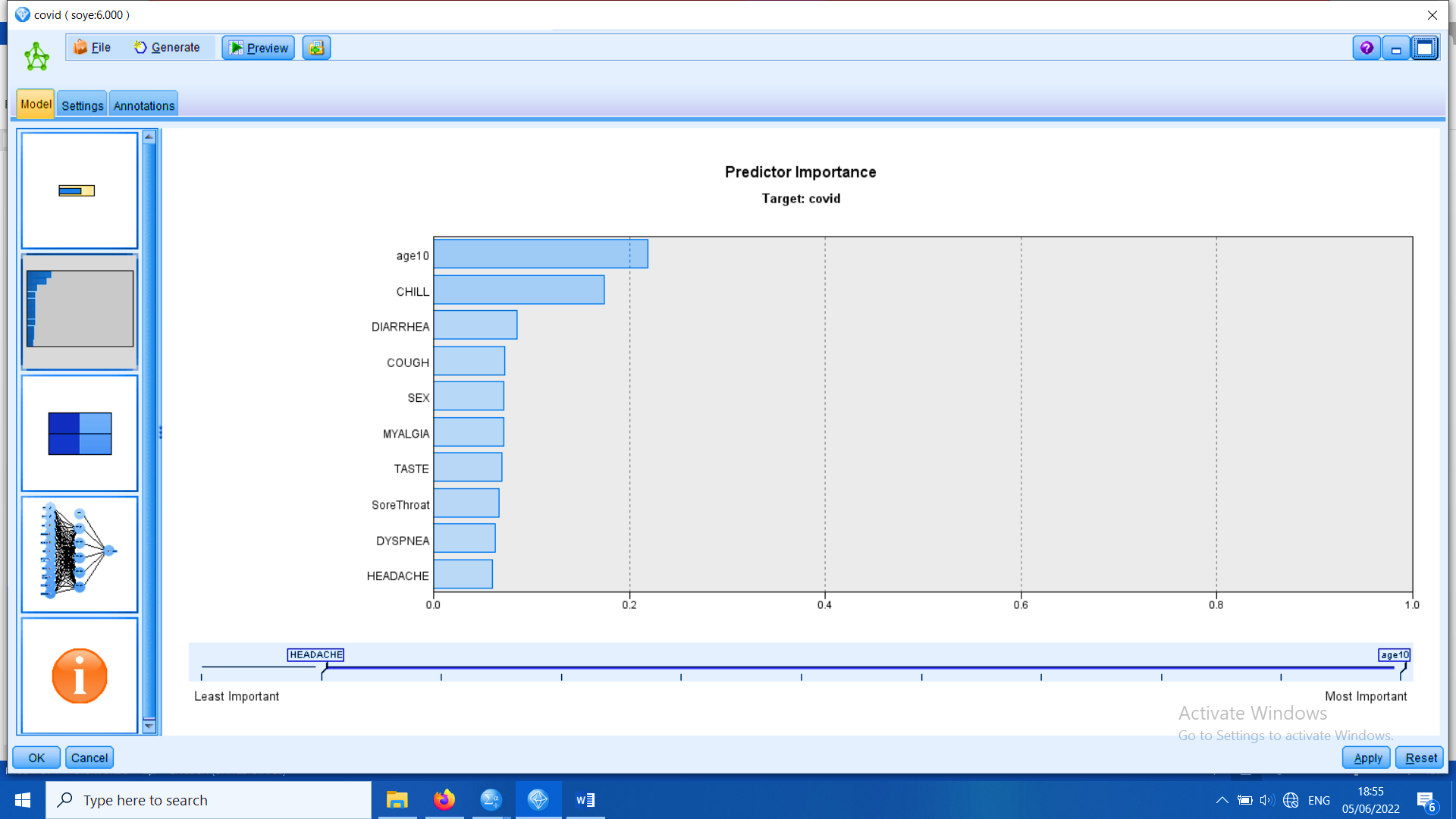 |
